# Locoregional Treatments for Bridging and Downstaging HCC to Liver Transplantation

**DOI:** 10.3390/cancers13215558

**Published:** 2021-11-05

**Authors:** Laura Crocetti, Elena Bozzi, Paola Scalise, Irene Bargellini, Giulia Lorenzoni, Davide Ghinolfi, Daniela Campani, Emanuele Balzano, Paolo De Simone, Roberto Cioni

**Affiliations:** 1 Division of Interventional Radiology, Azienda Ospedaliero Universitaria Pisana, 56126 Pisa, Italy; e.bozzi@ao-pisa.toscana.it (E.B.); paola.scalise@ao-pisa.toscana.it (P.S.); irene.bargellini@ao-pisa.toscana.it (I.B.); giulia.lorenzoni@ao-pisa.toscana.it (G.L.); r.cioni@ao-pisa.toscana.it (R.C.); 2 Department of Surgical, Medical and Molecular Pathology and Critical Care Medicine, University of Pisa, 56126 Pisa, Italy; daniela.campani@med.unipi.it (D.C.); p.desimone@ao-pisa.toscana.it (P.D.S.); 3 Division of Hepatobiliary Surgery and Liver Transplantation, Azienda Ospedaliero Universitaria Pisana, 56126 Pisa, Italy; d.ghinolfi@ao-pisa.toscana.it (D.G.); e.balzano@ao-pisa.toscana.it (E.B.); 4 Division of Pathology, Azienda Ospedaliero Universitaria Pisana, 56126 Pisa, Italy

**Keywords:** hepatocellular carcinoma, liver transplantation, loco-regional therapy

## Abstract

**Simple Summary:**

Liver transplantation is the first-line treatment for patients diagnosed with unresectable early stage hepatocellular carcinoma in the setting of cirrhosis. Patients with tumours beyond this stage may benefit from liver transplantation if their tumours are successfully downstaged. Loco-regional therapies, including ablation, trans-arterial and radiotherapeutical approaches are commonly used to treat patients before transplant, with the aim of reducing the risk of drop off from the waiting list due to tumor progression for patients within transplantation criteria as well as decreasing tumour dimension to meet acceptable criteria. In this review, current evidence on the safety, efficacy and utility of locoregional therapies as neoadjuvant therapies before liver transplantation are summarized.

**Abstract:**

Liver transplantation (LT) is the first-line treatment for patients diagnosed with unresectable early-stage hepatocellular carcinoma (HCC) in the setting of cirrhosis. It is well known that HCC patients within the Milan criteria (solitary tumour ≤ 5 cm or ≤3 tumours, each <3 cm) could undergo LT with excellent results. However, there is a growing tendency to enlarge inclusion criteria since the Milan criteria are nowadays considered too restrictive and may exclude patients who would benefit from LT. On the other hand, there is a persistent shortage of donor organs. In this scenario, there is consensus about the role of loco-regional therapy (LRT) during the waiting list to select patients who would benefit more from LT, reducing the risk of drop off from the waiting list as well as decreasing tumour dimension to meet acceptable criteria for LT. In this review, current evidence on the safety, efficacy and utility of LRTs as neoadjuvant therapies before LT are summarized.

## 1. Introduction

Liver transplantation (LT) is the first-line treatment for patients diagnosed with unresectable hepatocellular carcinoma (HCC) in the setting of cirrhosis. In 1996, Mazzaferro et al. provided seminal work showing that patients with HCC in the early stages could undergo LT with excellent results [1]. The Authors defined criteria for successful LT: solitary tumour ≤ 5 cm or ≤3 tumours, each <3 cm, now referred to as the Milan criteria. These criteria have been subsequently adopted by the United Network for Organ Sharing (UNOS) as the optimal criteria to select patients with HCC for LT [2]. When these selection criteria are applied, excellent overall four-year actuarial (75%) and recurrence-free survival (RFS) (83%) rates can be achieved [3]. 

However, the Milan criteria are nowadays considered too restrictive and may exclude patients who would benefit from LT. Several expanded criteria have been externally validated. Among them are the “UCSF criteria”, which include a single tumour up to 6.5 cm in diameter, or up to 3 tumours with the largest being 4.5 cm in diameter and a total tumour diameter of< 8 cm [4]; the Up-to-7 criteria (i.e., those HCCs having the number 7 as the sum of the size (cm) of the largest tumour and the number of tumours [5]; the AFP-French model (i.e., a points system based on tumour size, number of tumours and AFP cut-off levels at 100 ng/mL and 1000 ng/mL) [6]. AFP levels, size of the nodules and number of the nodules may be considered as continuous variables to predict the survival probability after LT, as proposed in the Metroticket Project, also available as an online calculator (http://www.hcc-olt-metroticket.org/, accessed on 5 September 2021) [7]. In this scenario, where on the one hand there is the tendency to enlarge inclusion criteria, while on the other hand there is the risk of donor shortage, there is consensus about the role of loco-regional therapy (LRT) during the waiting list to select patients who would benefit more from LT, reducing the risk of drop off from the waiting list as well as reducing tumour dimensions to meet acceptable criteria for LT [8]. 

Suitability and response to LRT has been considered, together with tumor stage, to stage patients and identify eight classes of transplantable tumours in a more “dynamic” manner with respect to the traditional “static” criteria described above [9]. This staging system has been validated by Di Sandro et al, who demonstrated that it can adequately describe the post-LT recurrence, especially in low-risk and high-risk class patients [10]. As a consequence, prioritization policies may be adapted. 

## 2. Bridging

‘‘Bridging” describes the treatment of accepted transplant candidates within Milan criteria while on the waiting list. During the waiting period for LT, patients with HCC are at risk of list-drop out due to tumour progression. Therefore, bridging therapy is recommended for patients with an estimated waiting time of ≥6 months. In fact, approximately 22% of patients with HCC drops off the liver transplant wait list. In half of these patients, this is due to tumour progression [11]. The effectiveness of LRT as neo-adjuvant therapies with a “bridging” intent has been demonstrated by several studies [12,13]. 

Response to bridging treatments may also influence not just drop-outs, but also the rate of post-transplantation tumour recurrences. However, the results of LRT on postoperative HCC recurrence are not well defined. Some studies found no difference between treated and non-treated patients [14,15], while in the series of Oligane et al., recurrence was significantly lower in the bridging LRT group compared to patients who did not undergo LRT [16]. The initial study of Ravaioli et al. showed that complete tumour necrosis induced by LRT decreased the rates of recurrence compared with partial necrosis [17], and it has been recently confirmed by other Authors. The analysis of tumour behavior on the basis of disease response to pretransplant LRT while patients are on the waiting list (complete pathologic response vs. partial response), seems to predict posttransplant outcomes such as HCC recurrence as well as RFS [18,19]. Di Norcia et al. made a comparison among patients receiving pre-LT LRT with and without complete pathological response from the United States Multicenter HCC Transplant Consortium (UMHTC) to evaluate how complete pathological response affects post-LT HCC recurrence and survival. Results showed that patients with complete pathological response had significantly lower cumulative incidences of HCC recurrence at 1, 3, and 5 years post-LT, compared with recipients without complete pathological response [20].

A large multicentric European study performed in patients treated with LRT showed that if ≥2 cm residual vital tissue is present at pathological analysis in the main lesion, this represents a strong independent risk factor for post-LT recurrence (hazard ratio [HR] = 5.6; *p* < 0.001) [21]. These results pair well with the data coming from an even larger multicentric study, based on 2103 HCC patients. Poor radiological response after LRT is one of the most important predictors for the risk of low intention-to-treat benefit after transplant [22].

LRT could therefore always be considered not only when the waiting time exceeds 6 months, and the goal of these treatments should be to obtain the best possible response. LRT protocols are very heterogeneous among centers, but ablation and trans-arterial therapies with different embolic platforms are almost universally used [23]. The multicentric study based on the European Liver Transplant Registry (ELTR) database, showed that HCC patients receiving LRT before LT had better 5-year survival rates with respect to no-LRT cases (69.7% vs. 65.8%; *p* < 0.001). At subgroups analysis, different numbers of treatments were related to differences in survival: one-two treatments showed improved survivals compared to no treatment (HR = 0.85 and 0.71, respectively), while no association was noticed if three or more treatments were needed (HR = 1.11), both in univariate and multivariate analysis [23].

All this evidence supports that, as already proposed by EASL in 2018, composite criteria that consider surrogates of tumour biology—among which AFP is the most relevant—and response to neoadjuvant treatments in combination with tumour size and number of nodules, are likely to replace conventional criteria for defining transplantability [8]. 

## 3. Downstaging

The term “downstaging” is intended for the treatment of HCC lesions in patients whose tumor burden is outside accepted transplantation criteria, with the aim to bring them within acceptable criteria and therefore allow them to achieve an expected survival after LT equal to patients who are within transplant criteria and do not need downstaging [8]. According to EASL guidelines, patients initially outside criteria are accepted as LT candidates only if their HCC is successfully down-staged to within Milan criteria [8].

Besides decreasing tumour burden, the relevant advantage of downstaging is that it allows time to select, among the treated patients, the ones with less aggressive tumor biology. Therefore, a thorough definition of the concept of downstaging must include both enlarged criteria and the results of LRT. Numerous inclusion criteria for downstaging have been developed; at UCSF, the down-staging protocol includes patients with one tumour ≤ 8 cm, two or three tumours each ≤5 cm and the sum of the maximal tumour diameters ≤ 8 cm, and four or five tumours each ≤3 cm and the sum of the maximal tumour diameters ≤ 8 cm [24,25]. The Italian Bologna group instead considers one tumour ≤ 6 cm, two tumours each ≤5 cm, and three to five tumours each ≤ 4 cm with the sum of maximal diameters ≤ 12 cm [26]. Other centers in the USA [27,28] apply UNOS T3 criteria (no upper limits in tumour diameter) as entry criteria. All patients considered for downstaging should have preserved liver function and good performance status so as to allow for LRT to be performed safely. According to these criteria, patients with radiographic evidence of tumour macrovascular invasion are excluded from downstaging [29]. 

Interestingly, even when downstaging therapies were applied with no upfront restriction, and successful downstaging was obtained, no differences in overall survival (OS) were demonstrated with respect to patients initially within Milan criteria [30].

Imaging is performed after LRT to restage the disease and it has been proposed that each tumour nodule should be defined as active if showing at dynamic radiological imaging (contrast-enhanced computed tomography (CT) scan or magnetic resonance (MR) imaging) an enhancement in the arterial phase with venous washout, even if this is only a part of an otherwise necrotic nodule. Therefore, fully necrotic HCC should count zero in such a prognostic computation. Conversely, each tumour nodule showing even a partial enhancement after neoadjuvant/downstaging treatment should be considered as totally vital (i.e., including in the tumour size calculation any concomitant necrotic area) [8,31]. The individual survival prediction by means of the Metroticket calculator may be performed after receiving neoadjuvant/downstaging treatment applying the above described criteria [7].

Similarly to the bridging scenario, the aim of LRT in downstaging is to obtain the maximum result in terms of response in each treated lesion. Due to the larger tumour burden of patients outside Milan criteria, therapies for downstaging include transarterial chemoembolization (TACE), transarterial radioembolization (RE) with yttrium-90 in the majority of cases.

## 4. Treatments for Bridging and Downstaging

Patients with compensated liver cirrhosis and small tumour size are preferably treated with a percutaneous approach, eventually associated with chemoembolization, whereas in patients with larger tumour burden and preserved liver function, an intra-arterial approach—that includes chemo- and radioembolization—is usually preferred.

### 4.1. Thermal Ablation

Among percutaneous therapies, radiofrequency (RF) ablation is a widely used technique, and its safety and efficacy are well established. In the setting of bridging to LT, the safety of RF ablation (RFA) has been proved by several studies since about twenty years ago, confirming the safety of the technique [31,32,33,34,35]. In these studies, the efficacy in terms of complete response on treated nodules were suboptimal, in the range of 41–66%. Probably at that time, when the importance of reaching complete response before transplantation was less evident, ablation was performed to control the disease rather than to eradicate the tumour, which may have influenced the results. 

More recently, Lee et al. retrospectively evaluated patients treated with RFA as a unique bridge technique before transplantation, reporting 72% of complete pathological response, rising to 79% in nodules below 3 cm in size. Of importance, a lower rate of complete necrosis of HCC was demonstrated in patients with post-LT HCC recurrence than in those without recurrence [36]. In a series of 125 HCCs treated with RFA only before LT, complete pathological response was observed in 61.6% on explanted livers, being 76.9% in nodules < 2 cm, 55.0% in nodules 2–3 cm, and 30.8% in nodules >3 cm. As in the series of Lu et al. [32], the importance of the “heat sink effect” pronounced for RFA was confirmed: tumours near hepatic vessels had complete pathological response of 50% versus 69.3% for tumours distant from vessels (*p* = 0.039) [37]. The role of RFA is also confirmed by the aforementioned study by Pommegaard et al, who investigated the effect of different LRT to improve survival after LT. Results showed that RFA was the one monotherapy with the strongest association with improved OS and HCC-specific survival, both in univariate and multivariate analyses, with beneficial effect also if used in combination to transarterial chemoembolization [HR 0.74 (0.55–0.99)] [23]. 

RFA has also been applied in potentially transplantable patients with HCC less than 3 cm in size [38]. The Authors reported 1-, 3- and 5-years actuarial survival rates after ablation of 98.2%, 86.2% and 79.0% in the HCC ≤ 2 cm group, vs.93.3%, 77.6% and 70.9% in the HCC >2 cm group (*p* = 0.01). When analyzing the pattern of recurrence, and in particular recurrence outside Milan criteria, tumour size > 2 cm (HR 1.94; 95% confidence intervals [CI] 1.25–3.02) and AFP at the time of ablation (HR 2.05; 95% CI 1.10–3.83) for AFP 100–1000 ng/mL were demonstrated to be significant prognostic factors. In a sensitivity analysis of patients who had tumour biopsies, poorly differentiated HCC was associated with an increased risk of recurrence beyond Milan criteria (HR 4.45; 95% CI 1.20–16.61). The Authors suggest that this group of patients should be considered immediately after the first HCC recurrence. The role of LRT to select patients according to tumour aggressiveness has been confirmed once more [38].

Nowadays, microwave (MW) ablation is increasingly employed as an alternative to RFA, thanks to the possibility to obtain larger volumes of necrosis in less time. There are not yet enough comparative studies to draw conclusions about the superiority of one of these techniques over the other. However, lesions abutting large vessels may respond better to MW ablation (MWA) due to the weaker influence of the “heat-sink” effect associated with the use of MW technology [39]. 

The Padua group retrospectively evaluated six patients who underwent MWA before LT either for bridging or downstaging. In all six cases, no peritoneal or nodal HCC macroscopic and microscopic diffusion was observed intraoperatively at the time of laparotomy for LT and no patient who underwent LT suffered any complication during or after the ablative procedure [40]. 

Som et al. [41] obtained histopathologic necrosis in 66% of cases at explanted livers in a series of 62 patients with HCC within Milan criteria, and treated with MWA as bridging therapy to subsequent LT. Even though they ablated tumours up to 4.6 cm, no significant predictors for incomplete necrosis were found, including tumour size. For those patients who underwent LT, survival was almost equivalent to whether or not complete tumour necrosis was found. Of interest, the study reported a longer median time to LT (10.9 months vs. 7.5 months) with respect to RFA studies [32,34,42,43,44]. These results hint that full tumour necrosis following MWA may not have a significant impact on survival, with the act of significant tumour debulking potentially being adequate to produce a robust survival effect [41].

In a recent retrospective study including 40 HCC nodules percutaneously treated with MWA in patients who subsequently underwent LT, complete and partial necrosis were found in 77.8% and 22.2% of cases, respectively, at the excised liver after LT [45]. These results were obtained with a single MWA session, whereas often in RFA series the treatment is applied two, or even three, times [36,37,45]. 

It would be interesting to confirm this data for MWA with more studies: due to the lower number of sessions, the different mechanism of action (friction of water molecule, fast acting, highest temperatures) and the lower inflammatory response in adjacent liver, MWA could produce a deeper and longer-lasting response.

### 4.2. Transarterial Chemoembolization

Transarterial chemoembolization (TACE) is the current first-choice treatment in patients with unresectable intermediate-stage HCC [8,46]. Over the last several years, TACE has been widely used as a bridge to transplant in patients with unresectable HCC and to downstage patients outside Milan criteria [23,47]. However, there is no agreement on how TACE should be performed, with high variability in terms of anti-cancer drugs and embolization modalities [48], without any clear demonstration of the superiority of a specific embolic or drug [49]. 

When TACE was performed as a bridging therapy before LT, large studies have shown dropout rates of 3–9.3%, which is lower than the rates before the use of bridging therapy [50,51]. Pretreatment with TACE was positively correlated with posttransplant survival, with patients having a 44% reduction in posttransplant mortality [52]. Sandow et al. retrospectively evaluated 142 consecutive patients with treatment-naïve HCC who were initially treated with TACE (both conventional and DEB-TACE) over a 12-year period, and who subsequently received a liver transplant. They showed that tumour biology (tumour grade and satellite nodules) and objective imaging response to TACE are associated with tumour recurrence after LT for HCC [53].

A prospective study recently published by Affonso et al. [54] included 200 HCC patients who underwent LT after TACE with drug-eluting beads (DEB-TACE) for downstaging versus bridging. They reported five-year posttransplant OS of 73.5% in downstaging and 72.3% bridging groups (*p* = 0.31), and RFS was 62.1% in downstaging and 74.8% bridging groups (*p* = 0.93), concluding that tumours initially exceeding Milan criteria and down-staged after DEB-TACE can achieve posttransplant survival and HCC recurrence-free probability, at five years, just like patients within Milan criteria in patients undergoing DEB-TACE [54].

**Figure 1:**
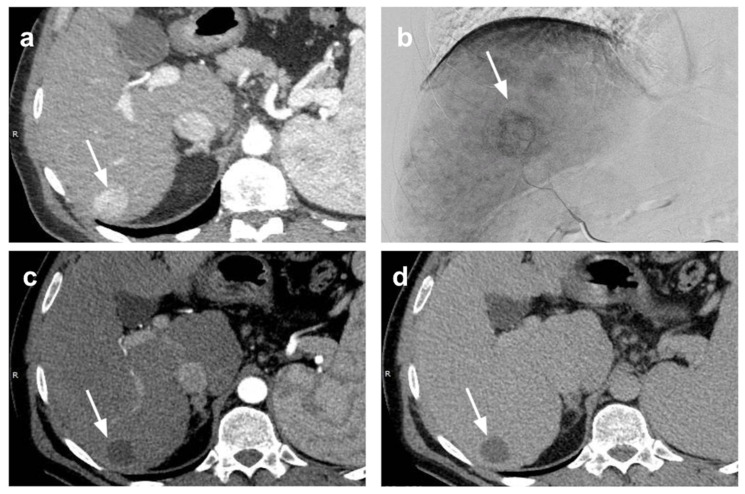

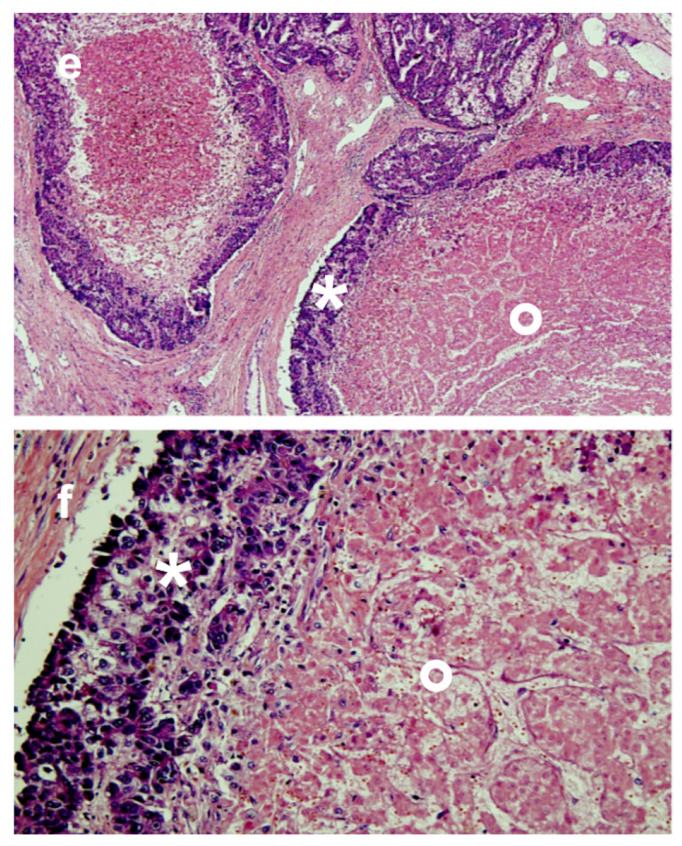
Bridging of HCC with TACE. Small HCC in hepatic segment 6 (arrow) at (**a**) pre-treatment CT scan in arterial phase and (**b**) at digital subtraction angiography. Complete radiological response was depicted at one-month CT evaluation after p-TACE (arrow) without appreciable enhancing tissue in (**c**) arterial and (**d**) portal-venous phase. The patient underwent liver transplantation two years after p-TACE. At pathologic examination of the explanted liver (**e**,**f**), extensive necrosis (*) was found in the treated area with presence of peripheral viable tumor tissue (ο) (**e**: magnification 20×; **f**: magnification 40×).

Even when TACE is performed as a neoadjuvant therapy before LT, there is not enough data to establish if it is preferable to use conventional TACE (c-TACE) or DEB-TACE. A recent publication demonstrates that, compared to lipiodol-TACE, DEB-TACE is better tolerated, allowing for reduced hospitalization, and is associated with more durable local tumour control after complete radiological response. These features may be of specific importance if applied to a patient during a possibly long waiting period before LT [55] (Figure 1).

Moreover, it is well known that post-TACE ischemia induces an increase in vascular endothelial growth factor (VEGF) plasma levels, potentially favouring tumour growth following neo-angiogenesis promotion [56]. VEGF plasma levels were significantly higher in a cohort of c-TACE patients until 28 days after c-TACE, compared to a cohort of DEB-TACE patients [57]. Thus, DEB-TACE may be preferable when TACE is used as neo-adjuvant therapy before LT. 

TACE can induce decompensation of cirrhotic liver, and therefore patient selection is crucial, especially in pre-transplant setting [58]. Therefore, absolute contraindications for TACE include decompensated cirrhosis (Child-Pugh B equal or higher than 8), extensive tumour with replacement of both lobes, technical contraindications to hepatic intra-arterial treatment, e.g., arteriovenous fistula, severely reduced portal vein flow, and renal insufficiency (creatinine > 2 mg/dL or creatinine clearance < 30 mL/min) [59]. Moreover, TACE should not be repeated when substantial necrosis is not achieved after two TACE treatments or when there is progression or liver function impairment or worsening of performance status (PS) [60].

Some concerns have been raised about the increase in posttransplant complications in patients previously submitted to TACE. TACE can cause damage to the inner lining or intima of the hepatic artery, potentially increasing the risk of hepatic artery thrombosis. A systematic review, representing 1122 patients from 14 retrospective studies, found that pre-LT TACE was significantly associated with the occurrence of posttransplant hepatic artery complications (odds ratio, 1.57; 95% CI, 1.09–2.26; *p* = 0.02). No significant association between neoadjuvant TACE and hepatic artery thrombosis was found [61].

Two recent retrospective studies conducted in large cohorts of transplanted patients who were previously submitted to intra-arterial therapies showed that the incidence of hepatic artery thrombosis was quite similar in those who had (1.3–2) or had not received TACE (2–2.4%, respectively). Furthermore, in contrast to the study of Sneiders et al., in these studies TACE did not affect arterial complications [62,63].

### 4.3. Combined Treatments

Both thermal ablation and transarterial chemoembolization as monotherapy have demonstrated limitations, such as incomplete tumour necrosis, tumour recurrence, and inadequate control of medium to large size HCC. Previous studies have demonstrated the increased OS and RFS if these techniques are applied in combination, particularly for lesions larger than 3 cm [64,65,66].

TACE plus RFA performed better when compared with TACE alone and RFA alone, in a recent meta-analysis by Jiang et al. [63] including twenty-one studies involving 3413 patients. TACE plus RFA showed better OS (HR = 0.62, 95% CI = 0.55–0.71, *p* < 0.001) and RFS (HR = 0.52, 95% CI = 0.39–0.69, *p* < 0.001) than TACE alone; similarly, TACE plus RFA showed longer OS (HR = 0.63, 95% CI = 0.53–0.75, *p* < 0.001) and RFS (HR = 0.60, 95% CI = 0.51–0.71, *p* < 0.001) compared with RFA alone, even in patients with HCC larger than 3 cm [67].

These findings are in line with the results of a previous meta-analysis by Wang et al. who evaluated six studies with 534 patients, showing that the combination of TACE and RFA is associated with a significantly longer OS (HR = 0.62, 95% CI: 0.49–0.78, *p* < 0.001) and RFS (HR = 0.55, 95% CI: 0.40–0.76, *p* < 0.001) in contrast with RFA monotherapy, without significant difference in major complications [68]. The combination of RFA followed by DEB-TACE allowed to obtain sustained local control of the disease in patients with a single HCC > 3 cm, with better results in terms of CR (62.5% of treated lesions), 2-year cumulative HCC recurrence rate (48.1%) and OS rate (91.1%) respect to DEB-TACE alone [69].

According to the European Liver and Intestine Transplant Registry (ELITA), RFA plus TACE are applied as neoadjuvant therapies in about 8% of patients [23]. In a retrospective study, Vasnani et al. evaluated the histopathologic efficacy of DEB-TACE combined with percutaneous thermal ablation in patients bridged to LT. Combination therapy DEB-TACE/RFA versus DEB-TACE/MWA as a bridge to LT, demonstrated equivalent tumour coagulation in the absence of tumour seeding along the ablation tracts [70].

### 4.4. Transarterial Radioembolization

Y90 Radioembolization (RE) has emerged over the past decade as a locoregional treatment with favorable efficacy, safety profile, and quality-of-life outcomes [71,72]. The PREMIERE trial demonstrated in 2016 that Y90 RE prolongs time to progression (TTP) when compared to lipiodol-TACE for early–intermediate stage HCC (>26 months vs. 6.8 months, *p* < 0.01), suggesting more complete treatment of targeted lesions and tumour control. Improved tumour control could then potentially lower dropout rate from transplant listing [73]. 

Gabr et al. [74] conducted an intention-to-treat analysis of 362 patients with T2 HCC treated over a 15-year period. Even though all patients met Milan criteria, only 160/212 patients who had been judged eligible for listing were offered LT. About 5% of patients experienced waiting list dropout due to disease progression or death. All the endpoints of the study (OS, RFS, disease-specific mortality and time-to-recurrence) were affected by the extent of pathologic necrosis, with complete/extensive necrosis being associated with better OS compared to partial necrosis. Y90 RE appeared to provide a high degree of disease stability/response, usually achieved by one treatment, and resulting in few progressors. Favorable OS (67.5 months) was appreciable even in patients who did not undergo LT for any reason, in particular those with Child Pugh A disease [74].

A recent retrospective study compared posttransplant outcomes in patients undergoing bridging with Y90 RE and with TACE. Not surprisingly, significantly fewer treatments allowed maintaining patients within Milan Criteria in the Y90 RE group (1.46 vs. 2.43; *p* = 0.001), while there was no difference in time on the transplant list between the two groups [75]. In addition, microvascular invasion at histopathology, which represents a well-established prognostic factor associated with worse disease-free survival and OS, occurred significantly less in the Y90 RE group compared with the TACE group (3.6% vs. 27%; *p* = 0.013) [75,76]. Due to the small size of the Y90 particles (30 to 60 μm), tumour cell death primarily derives from radiation delivery causing apoptosis of endothelial cells [77]. Therefore, Y90 RE may be preventing tumour microvascular invasion by preferentially inducing apoptosis of the endothelial cells of small tumour neovessels.

This data raises the interesting possibility that Y90 RE may affect tumour biology and microenvironment in a unique way, which may further decrease posttransplant recurrence of HCC.

A landmark study demonstrating the positive impact of Y90 RE in downstaging HCC to LT was published in 2009 [27]. In this retrospective analysis comparing radioembolization with Y90 and TACE, successful downstaging from T3 to T2 was obtained in 58% of patients with Y-90 and 31% with TACE (*p* = 0.023). Event free survival also favored Y90 RE (17.7 vs. 7.1 months, *p* < 0.01). 

As reported by the single-center retrospective review by Ettorre et al., Y90 RE allowed the successful downstaging of about 80% of T3 patients to T2 and to bridge to LT all patients who previously were within Milan criteria [78]. Gabr and colleagues [74] showed similar results in one of the largest single-center experiences with Y90 RE prior to LT, with successful downstaging from T3 to T2 and bridging to transplant rates of 47% and 98%, respectively.

In this large series, no difference has been confirmed between RFS after Y90 RE and LT patients bridged versus those downstaged or within versus beyond Milan criteria [74].

When used as initial downstaging therapy, TACE and Y90 RE showed similar performance [79]. The comparison of the two techniques did not show any significant differences in terms of efficacy and downstaging rate (both more than 80%) as reported by the recent MERITS-LT multicenter study. However, it seems that Y90 RE allows better tumour local control to be achieved, since more completely necrotic tumour(s) (30.8% vs. 20.5%) and less tumours beyond Milan criteria (23.1% vs. 43.2%), as well as microvascular invasion (7.7% vs. 20.5%; all *p* > 0.25), were found at histopathological analysis of the explanted livers in the Y90 RE group [79].

The safety of Y90 RE in a pretransplant setting, in particular regarding the risk for hepatic artery dissection during hepatectomy, hepatic artery thrombosis, and a propensity for anastomotic stenosis or pseudoaneurysm formation has been assessed. A retrospective study showed that neither radioembolization nor chemoembolization appears to increase the risk of peritransplant hepatic arterial complications [63]. Additionally, minimizing lung shunting and preserving lung function before an intensive liver transplant procedure may be of paramount importance. In some studies performing RE as a bridge to transplantation, none specifically mentioned pulmonary complications after LT [80,81]. 

Radioembolization may also be performed with a Holmium-166 based platform. Besides offering peculiar imaging capabilities, the short half-life of Holmium-166 ensures delivery of a high dose rate (90% of radiotherapy dose delivered within 4 days). Clinical evidence demonstrates that Ho-166 RE is efficacious, well tolerated, and safe for the treatment of unresectable liver cancer [82,83,84].

**Figure 2:**
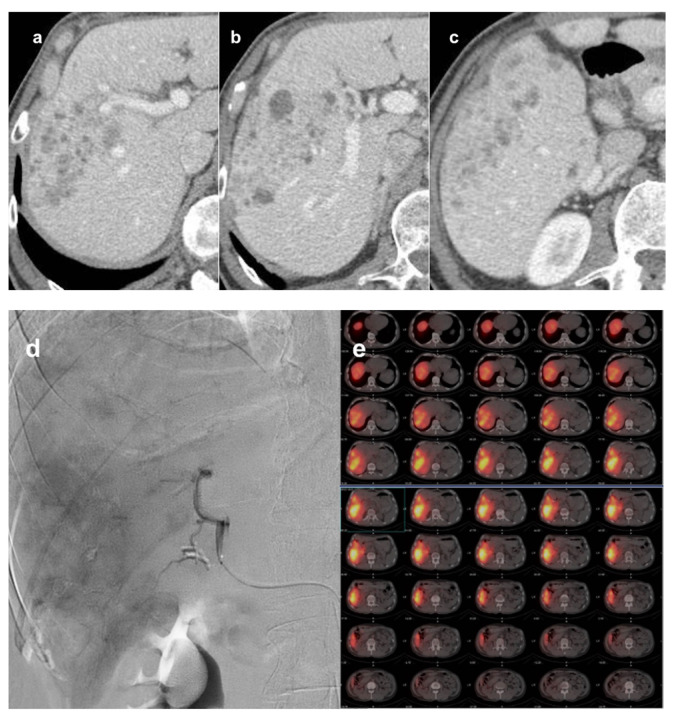

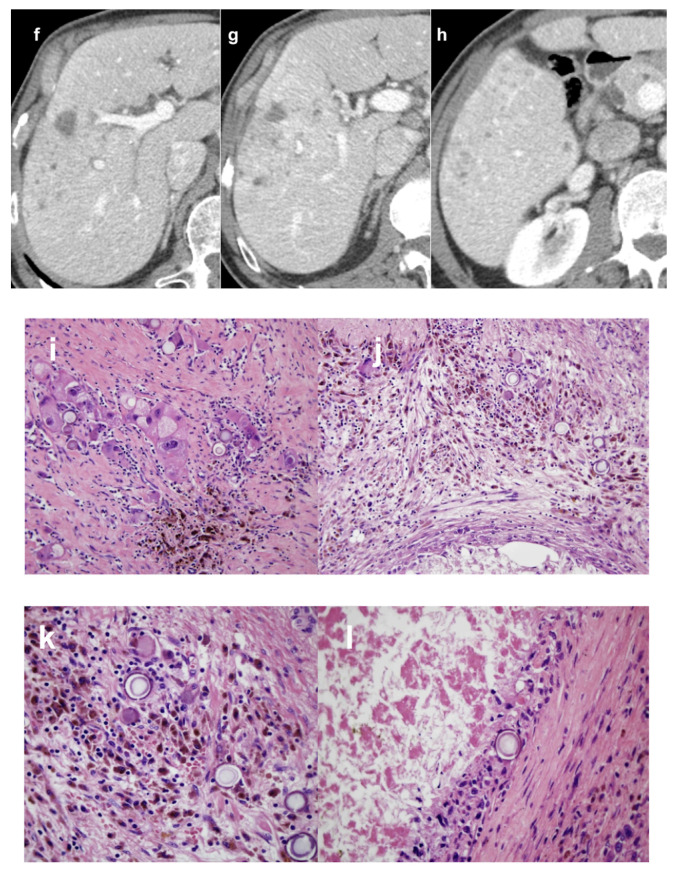
Downstaging of HCC with ^116^Holmium TARE. Infiltrative biopsy proven HCC in right liver lobe at CT before treatment (**a**–**c**, different levels) in portal-venous phase; AFP was 493 μg/L. ^116^Holmium TARE was performed with a downstaging intent (**d**: angiogram from the right hepatic artery; **e**: PET-CT after treatment); the administered activity was 3.6 GBq. At 45 days CT follow-up (**f**–**h**, same levels as **a**–**c**), lesion progressive devascularization and decrease in size were depicted; AFP was 251 μg/L. Liver transplantation was performed 81 days after ^116^Holmium TARE. At pathologic examination of the explanted liver (**i**–**l**), ≈ 50% necrosis was found with viable tumor mainly at the periphery of the lesion and fibroblastic reaction associated with inflammatory response rich in pigmented macrophages. ^116^Holmium microspheres are visible in histology (**I**,**j**: magnification 20×; **k**,**l**: magnification 40×).

Ho166 RE has not yet been explored in the setting of pretransplant patients, but physical properties of this specific RE platform may translate to a quicker response after RE with respect to Y90, and patients included in downstaging protocols may particularly benefit from this feature (Figure 2).

### 4.5. Radiotherapeutic Approaches

In recent years, high conformal high dose rate (HDR) brachytherapy and stereotactic body radiotherapy (SBRT) have evolved as alternatives to thermal ablation when ablation is not safe or technically feasible, as they are not limited by adjacency to large vessels, exophytic growth or central location [85]. They have been applied in a small cohort of 14 patients not amenable for thermal ablation, as a bridging therapy before LT. No viable tumor was found in 8 of the 12 available liver specimens. One patient experienced a grade 3 bleeding after the removal of the catheter used for brachytherapy and one patient underwent liver failure within 3 weeks after treatment [86]. These experiences follow the publication of several small series where only SBRT was performed with different dose fractionations and therefore their results are difficult to interpret. In a recent large cohort study, SBRT has been performed to bridge patients not eligible for other LRTs, and it was demonstrated to be as safe and effective as TACE or RF ablation [87]. Finally, a prospective trial about the use of SBRT as primary bridging modality—as compared with historical cohorts of patients treated for TACE or high focused ultrasound (HIFU)—has been published. SBRT was demonstrated to be safe, with a significantly higher tumor control rate and reduced risk of waitlist dropout. The Authors concluded that SBRT should be used as an alternative to conventional bridging therapies [88]. 

The safety and effectiveness of the combination of TACE and SBRT has also been evaluated in the bridging scenario. The rate of complete necrosis in liver specimens was higher in the combination therapy group (8 out of 9 patients, 89%) with respect to the TACE (0 of 14 patients, 0%) and SBRT (1 of 4 patients, 25%) groups [89]. The low sample size and retrospective nature of the study do not allow definitive conclusions to be drawn about the role of this combination therapy, which needs to be investigated in further studies. 

## 5. Future Prospects

Because of the continuous development of therapy options and positive results of their application, an individualized bridging/downstaging therapy is possible. In this scenario, a consensus on expanded criteria for LT in HCC has not been reached. In particular, given the limited pool of transplantable organs, further validation of good outcomes of more advanced stage patients downstaged using LRT is necessary. Chapman et al. present 63 cases of HCC beyond the Milan criteria who underwent LT after successful downstaging to within the Milan criteria. They compared the results with patients initially within the Milan criteria, and the results showed that aggressive attempts at downstaging, without a priori exclusion, allows for excellent long-term results similar to patients presenting with earlier stage disease [30]. 

Even though portal vein thrombosis has been traditionally considered a major contraindication to LT, due to the high recurrence rate, there were few studies reporting the survival outcomes of LT for HCC patients with PVTT [81,82]. The 5-year OS ranged from 50.3–63.6% [90,91]. More recently, the Hong Kong group confirmed good outcomes in HCC patients with PVTT involving the lobar or segmental level (55% survival at 5 years, HCC recurrence in 50%) [92]. A modest expansion of selection criteria to include small HCC with segmental PVTT has been therefore advocated. 

In this perspective, Levi Sandri et al. treated with Y90-RE four patients of HCC with portal vein tumour thrombosis (PVTT) and initial BCLC-C classification due to macrovascular invasion. All patients were successfully downstaged and underwent LT within the Milan criteria, obtaining an OS of 39.1 months without any recurrence or death [93]. In a recent retrospective study by Assalino et al., selected HCC patients with radiological signs of vascular invasion could be considered for transplantation, provided that they previously underwent successful treatment (with RE, TACE or surgery) of the macrovascular invasion resulting in a pretransplant AFP < 10 ng/mL, with an expected risk of posttransplant HCC recurrence of 11% [94].

In the regions of organ shortage, deceased liver graft is not allocated to HCC patients with portal vein thrombosis in view of the anticipated worse outcomes compared with HCC patients without neoplastic thrombosis. In this scenario, expanding the organ donor pool beyond the currently accepted criteria is the current focus of exciting and fruitful research. Machine perfusion has expanded in the last few years due to its capacity to preserve grafts in quasi-physiological conditions before implantation, reduce cold storage–related injuries, and assess graft function prior to transplantation. In addition, machine perfusion can be combined with organ repair and reconditioning, thus reaching the goal of preserving organs after circulatory death and recovering organs otherwise not acceptable [95]. Successful downstaging of HCC by means of locoregional treatments and expansion of donor pools with machine perfusion may be the keys for improving oncological results even in selected patients with advanced HCC.

## 6. Conclusions

The role of LRTs to bridge or downstage patients with HCC to LT is well-established and performed at all transplantation centers. Selection of patients should be done in a multidisciplinary setting evaluating, not only patients’ tumor burden, liver function and general conditions, but also the expected time on the waiting list and transplant benefit for the single patient. Among different LRTs the choice to perform percutaneous, trans-arterial or external radiotherapeutic approaches is then undertaken according to size, number or location of HCCs. Overall, LRTs are effective both in bridging patients to LT reducing drop-out rates during waiting time, and in downstaging. In particular overall survival rates in patients successfully downstaged within Milan, are similar to those that are initially within Milan criteria for LT.
